# Automated Distinction between Cement Paste and Aggregates of Concrete Using Laser-Induced Breakdown Spectroscopy

**DOI:** 10.3390/ma14164624

**Published:** 2021-08-17

**Authors:** Pakdad Pourbozorgi Langroudi, Gesa Kapteina, Marcus Illguth

**Affiliations:** Civil Engineering Department, The University of the Built Environment and Metropolitan Development, HafenCity Universität Hamburg (HCU), Henning-Voscherau-Platz 1, 20457 Hamburg, Germany; gesa.kapteina@hcu-hamburg.de (G.K.); marcus.illguth@hcu-hamburg.de (M.I.)

**Keywords:** LIBS, element distribution, machine learning, classification, concrete, heterogeneous materials

## Abstract

Laser-induced breakdown spectroscopy (LIBS) is a technique which enables the analysis of material components with precision and spatial resolution. Furthermore, the investigation method is comparatively fast which enables illustrating the distribution of elements within the examined material. This opens new possibilities for the investigation of very heterogeneous materials, such as concrete. Concrete consists of cement, water, and aggregates. As most of the transport processes take place exclusively in the hardened cement paste, relevant limit values linked to harmful element contents are specified in relation to the cement mass. When a concrete sample from an existing structure is examined, information on the concrete composition is usually not available. Therefore, assumptions have to be made to convert the element content analyzed in the sample based on the cement content in the sample. This inevitably leads to inaccuracies. Therefore, a method for distinction between cement paste and aggregates is required. Cement and aggregate components are chemically very close to each other and therefore, complex for classification. This is why the consideration of a single distinguishing feature is not sufficient. In this paper, a machine learning method is described and has been used to automate the distinction of the cement paste and aggregates of the LIBS data to receive reliable information of this technique. The presented approach could potentially be employed for many heterogeneous materials with the same complexity to quantify the arbitrary substances.

## 1. Introduction

Laser-induced breakdown spectroscopy (LIBS), also sometimes called laser-induced plasma spectroscopy (LIPS), is an analytical atomic spectrometry technique which identifies elemental compositions of a material from its plasma emission. The plasma is formed with a high-power laser beam focused on the surface of the sample. The high thermal energy in the focal point brings the atoms to an excited state through evaporating and atomizing the sample and generate the plasma. When the atom relaxes to ground state, photons reveal the atoms’ unique identity [1,2]. At this state, the spectrometers disperse the emitted radiation of the laser-induced plasma to capture a spectrum in terms of intensity as a function of the wavelength [2]. LIBS developed rapidly in the past two decades as an analytical technique in order to identify and quantify elements and substances in materials [2]. It has a vast field of operation including planetary exploration-lander missions, industrial quality assurance, archaeology, environmental monitoring, and biological identification [1,3,4,5,6]. In civil engineering, LIBS has been used for the determination of chloride distribution within the concrete cover.

The determination of chloride content in reinforced concrete structures is a very important component in the field of maintenance and vulnerability analysis of existing reinforced concrete buildings. Chlorides can reach a building component, if exposed to deicing salt or sea water. Then they are able to penetrate into the concrete and over time may reach the reinforcement. If a critical chloride content is exceeded at the reinforcement bar, it may start to corrode. This has led to extensive damages and repair in the past. A reference value for this critical chloride content is defined as 0.5 wt.%/cement [7]. Therefore, a precise determination of the chloride distribution within the concrete cover of existing buildings may have a huge impact on the proposal of retrofit solutions or the residual service life calculated on the basis of these test results. To determine the chloride content, usually concrete powder is extracted from the structure by drilling. These powder samples are then further examined in the laboratory. Common approaches to measure chloride content are potentiometric titration, direct potentiometry, and photometry, which are all advised by the European Standards [8,9,10,11]. However, with these analytic methods no additional differentiation between cement and aggregate is possible. Hence, the results are related to the whole sample mass being concrete, but as explained before the chloride concentration needs to be related to cement. Therefore the analysis values have to be converted from wt.-%/concrete into wt.-%/cement, which requires the knowledge of the concrete composition in the analyzed concrete powder samples. Since this information is rarely available, assumptions for a conversation factor have to be made. This applies to the majority of cases where concrete samples are taken from existing structures. Due to this procedure the result in the decisive unit may be inaccurate although the analysis method itself is very precise. By excluding the aggregate within the examinations process, more reliable values for the chloride content in cement would be achievable. This would lead to optimized repair scopes and to more accurate estimates of the remaining service life, since the estimated remaining service life is based on the distribution of chlorides within the concrete cover.

The problem in identifying the aggregate and further distinguishing it from the hardened cement paste is due to the fact that the aggregate itself is very heterogeneous. In general the contained minerals and thus the chemical elements of the aggregates depend on the geology of the surrounding landscape where the concrete factory is located. Table 1 illustrates oxides in different types of aggregates and Portland cement (CEM I). On the basis of the element distribution in different minerals of which aggregates may consist in comparison to CEM I, it may not be possible to distinguish aggregates from cement with regard to the presence of a single element (see Table 2).

Therefore, a fast measuring method is required for simultaneous multi-element detection. With LIBS it is possible to scan a surface and provide an element distribution of the scanned surface. This is one of the fundamental advantages of LIBS over conventional methods. Despite the capabilities of the LIBS method, further processing is needed to identify aggregate in concrete. The data acquired by LIBS is usually large and the higher spatial resolution of the measurement leads to a higher amount of data and longer processing time to translate it to information. To speed up the processing and increase the accuracy in aggregate classification, an application of machine learning (ML) has been used to identify the aggregates.

## 2. Materials and Methods

### 2.1. LIBS Setup

**Figure 1:**
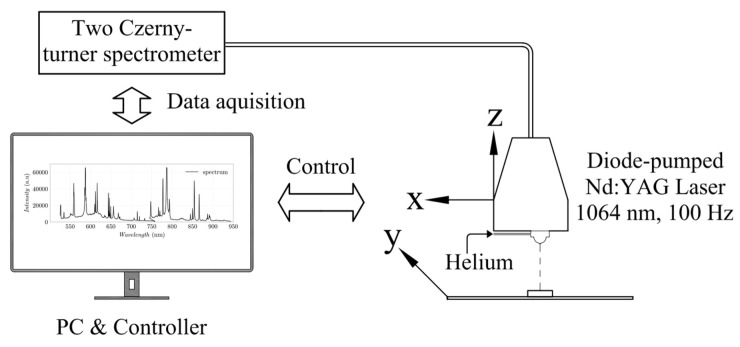
Schematic of LIBS general components.

LIBS’ main experimental instruments include lasers, spectrometers, detectors, and computers, as shown in Figure 1. The LIBS experimental setup used in this study consist of a diode-pumped Nd:YAG Laser with a wave length of 1064 nm, a pulse rate of 100 Hz, and an energy of 3 mJ (supplier Secopta FiberLIBSlab). This test setup is structurally comparable to other studies with a similar focus [16]. The laser setup leads to a power density of about I ≈ 39 GW/cm^2^ to form the plasma. The laser beam is focused to a spot size of about $d_{spot}\approx 80$ μm with confocal mirrors with a focal length of f ≈ 75 mm. The whole optical arrangement is mounted on a measurement head which has a typical working distance to the specimen of about 35 mm. A single optic has been used for laser and capturing the emission of the plasma radiation (see Figure 1). For detecting the emission signals a set of two Czerny-turner spectrometers in the ranges of near infrared (NIR) and visible (VIS) have been used. These spectrometers have the specification of 1200 lines/mm grating and 25 μm entrance slit (AvaSpec-ULS2048CL, Avantes, Apeldoorn, The Netherlands). The cover range of the VIS and NIR spectrometers are respectively 525–750 nm and 745–940 nm. The spectral resolution of the spectrometers are about δλ≈0.1 nm. The scanning intervals have been set to 0.1 mm × 0.1 mm steps for x and y dimension with an alternating movement on the z axis which is required for auto focusing of the laser spot. With this setup the element distribution on a sample can be determined two-dimensional. The measurement spot is purged with helium to eliminate the spectral lines of the gases, which are included in the air (mainly argon). Furthermore, helium intensifies the atomic line of chlorine @837.6 nm.

### 2.2. Samples

**Figure 2:**
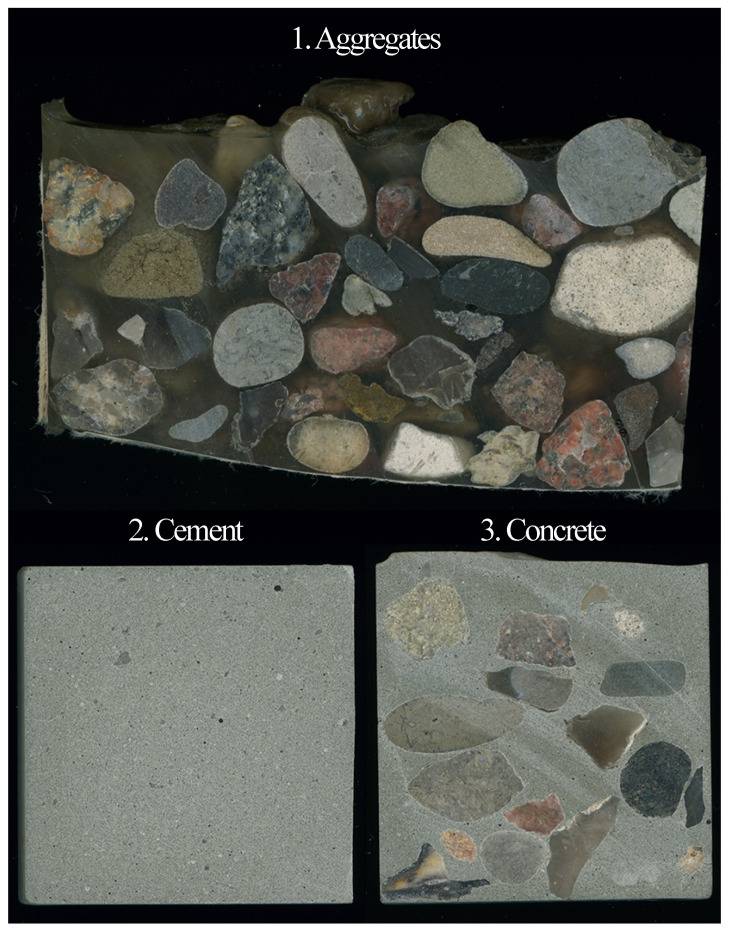
The three sample types used for experiments.

For investigation three sample types have been prepared with different mixtures leading to a set of 15 samples in total (see Figure 2). The sample types in connection with their investigation targets are given as follows:

Type 1 Aggregate specimen, comprised of aggregates and epoxy resin to exclusively characterize the aggregate and its variation by selected element distributions. Aggregates from various types with grain size in a range of 8–16 mm were filled in a paper cup and epoxy-resin was added to cover the gravels. Epoxy is a homogeneous polymer which similar to concrete contributes to the stabilization of the aggregates in the mixture. The homogeneity and the chemical composition of the epoxy resin allows an easy differentiation of the analyzed data in terms of epoxy or aggregate. This provides the basis for an adjusted data set. Due to this approach it was possible to investigate the different types of aggregate simultaneously, which facilitated the analysis. The used aggregates are classified as pyrogenic rock, migmatite, and gneiss with the main minerals feldspar (plagioclase), quartz, and mica.
Type 2 Cement specimen with Portland cement (CEM I 42.5 R) prepared with deionized water and a water-cement ratio of 0.50 to characterize solely the hardened cement paste by its element distributions.
Type 3 Concrete specimen with cement and water-cement ratio as described for the sample type (2) together with the aggregate measured within specimen type to verify the trained algorithm.

The sample types (1) and (2) have been used to characterize a virtual concrete whereas sample type (3) was used to verify the trained model in real application.

In order to show what results can be obtained with the method presented here, a drill core taken from an existing structure was also examined (in situ sample). The concrete core had been taken from a part of a structure, which had been exposed to deicing salt that contains chlorine. Before the measurement of this sample the core was split parallel to its axis to examine how far and to what extent chlorides have penetrated into the concrete.

### 2.3. Measurements

After curing, all test specimens were cut into slices and the cut surfaces were analyzed via LIBS. The slices with aggregate and epoxy resin (sample type (1)) were scanned over an extended area of 60 × 40 mm^2^ to obtain a higher number of data points to detect the potentially large scatter due to the various aggregate types. The slices of the specimen belonging to samples types (2) and (3) have been measured in an area of 35 × 35 mm^2^.

The measurement was performed in while motion mode. That means that the measurement head continuously moves with a specific speed over the surface of specimen. The speed of the measurement head is adjusted internally in the measurement system so that with a repetition rate of 100 Hz the mentioned grid size is met.

Major minerals that form the aggregates of concrete are: plagioclase, quartz, hematite, magnetite, amphibole, pyroxene, olivine, mica, and calcite. The element distribution in these minerals among each other, as well as compared to cement, have a lot similarities. With data taken from [17] the mass quantities for particular elements were calculated for those minerals (see Table 2). Because most minerals occur in several variations, the mass distribution of the bound elements is not fixed. Therefore, for each element a range (min and max) of the quantity is given. For the distinction between aggregates and cement, particular elements must be selected, which differ substantially in their amount when comparing these two materials. A useful element for this purpose is calcium. As can be seen in Table 2 almost all minerals are lacking calcium or have significantly lower amounts compared to cement, except limestone and pyroxene. As limestone is a very common mineral, calcium cannot be used as a single indicator. Chemically bound hydrogen could not be found in the majority of minerals and only in small quantities in amphibole, which are negligible. Differently, hardened cement paste binds large quantities of hydrogen, due to the hydration process taking place while hardening. However, hydrogen can also be present in form of physical water within the cement matrix as well as in porous aggregates such as limestone. Thus, information of the quantity of another element is needed. Beside calcium and oxygen, limestone consists of carbon, hence it has also been selected for the distinction. However, it is worthwhile to mention that the even though the Portland cement does not contain carbon in a substantial amount, during time the carbon content could rise due to the process of carbonization, which is a chemical reaction of ambient air with the concrete. In addition, there are further cement types available with varying ingredients, so that carbon alone isn’t a reliable indicator for distinction.

The spectral lines for the selected elements H, Ca, C, and O (for normalization, see Section 3.1) are taken from the National Institute of Standards and Technology (NIST) atomic spectra database [18]. The selected wavelengths with regard to available spectral ranges and experimental setup are given in Table 3.

The emission lines were selected in a way that self-absorption, over-saturation, and overlapping with other elements lines can be excluded. These criteria helped to select these wavelengths for further processing. For some elements like oxygen or calcium there is also the possibility to use some other lines. Some elements like silicon, iron, and titanium could be detected at shorter wavelengths in range of near ultra violet (NUV) and ultra violet (UV) but due to limitation of the control box of the experimental setup, it was not possible to cover this entire spectral range at the same time.

### 2.4. Machine Learning

In classical programming a rule is being defined and data is provided as input. With this information processing takes place under defined rules leading to the answer, which is the output. In machine learning (ML) there is a shift in this paradigm. An answer replaces the rule in order to have a rule as an output [19]. The different theories and forms of ML are explained in [19,20,21]. The data type, whether it is labeled or not and whether it is continuous or discrete, is decisive for a decent ML approach. Within this study the applicability of five well know algorithms have been examined, which are: Logistic Regression (LR), Decision Tree Classification (DTC), Ridge Classifier (RDG), Random Forest Classifier (RFC), and k-Nearest Neighbor (kNN). For all five algorithms, data from the model training procedure were evaluated and compared (see Section 3.2). It turned out that in the considered case the kNN algorithm showed the best performance, whereas it was chosen to solve the described task. The k-Nearest Neighbor (kNN) algorithm is a robust and versatile classifier and often used as a benchmark in more complex classifications such as artificial neural networks (ANN) and support vector machines (SVM) [22]. Despite its simplicity, kNN can compete with other classifiers and is applicable in a variety of analysis such as economic forecasting, data compression, and genetics. In terms of accuracy, the kNN algorithm competes with the most accurate models and it produces highly accurate predictions. Therefore, the kNN algorithm can be used in applications that require a high accuracy but not human-readable models [23]. Similarity or distance measures are the main components in distance-based clustering whereas the quality of the predictions relies on the distance measure. Thus, the KNN algorithm is suitable for applications where sufficient domain knowledge is available [23]. The kNN algorithm is a lazy learner, which means that no model is explicitly being learned. Rather, training instances are registered, which are then subsequently used as "knowledge" in the prediction phase. In practice, this means that it makes only a query from the database about the inquired label [22]. Although this approach increases computational costs in comparison to other algorithms, *kNN* is still the better choice for applications where accuracy is in higher priority and predictions are not requested frequently [23].

*kNN* is a non-parametric classifier, which means that the model has a fixed number of parameters [23]. This algorithm simply finds the *K* nearest points, to the input *x*, in the training set with the Euclidian distance. Then the members of each class within this set are counted and a vote is raised based on empirical fraction to estimate and predict. More formally,

(1) $$kNN:\;p\left(y=c|x,D,K\right)=\frac{1}{K}\sum_{i\in N_{K}\left(x,D\right)}I\left(y_{\left(i\right)}=c\right)$$

where $N_{K}\left(x,D\right)$ are the (indices of the) *K* nearest points to *x* in *D* and I(e) is the indicator function defined as follows:

(2) $$I\left(e\right)=\left\{\begin{array}{c} 1\;\mathrm{if}\;e\;\mathrm{is}\;\mathrm{true}\\ 0\;\mathrm{if}\;e\;\mathrm{is}\;\mathrm{true} \end{array}\right.$$

## 3. Results

### 3.1. Data Evaluation

**Figure 3:**
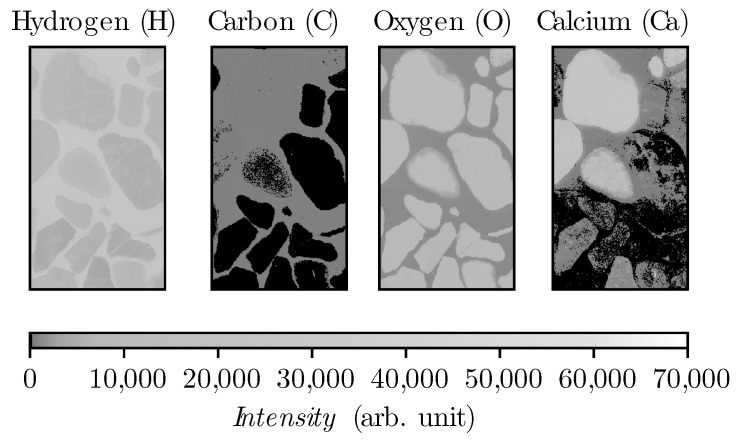
Heatmap from epoxy specimen in spectral range 525–940 nm.

The spectrometers disperse the emitted radiation of the laser-induced plasma to capture a spectrum in terms of intensity as a function of the wavelength [2]. In this study, line intensities have been used for signal evaluation. The signal intensities measured by LIBS show deviation over time due to various environmental conditions such as room temperature, spectrometer temperature, etc. Furthermore, in continuous use a decay of signal intensity occurs due to sand blast effect on laser protection lens. Therefore, an internal normalization is required to grantee the comparability of the measurements with each other. Hence, a ratio of the other elements with oxygen is used. Oxygen is suitable because this element is present in cement as well as in all types of aggregates (see Figure 3) and also has the lowest standard deviation compared to hydrogen (see Table 4). Therefore, oxygen is selected as the denominator and the other elements as numerators. Thus, three new sets of data have been created for each specimen considering the respective element ratio linked to oxygen. This procedure leads to the following data sets: H/O, C/O, and Ca/O.

**Figure 4:**
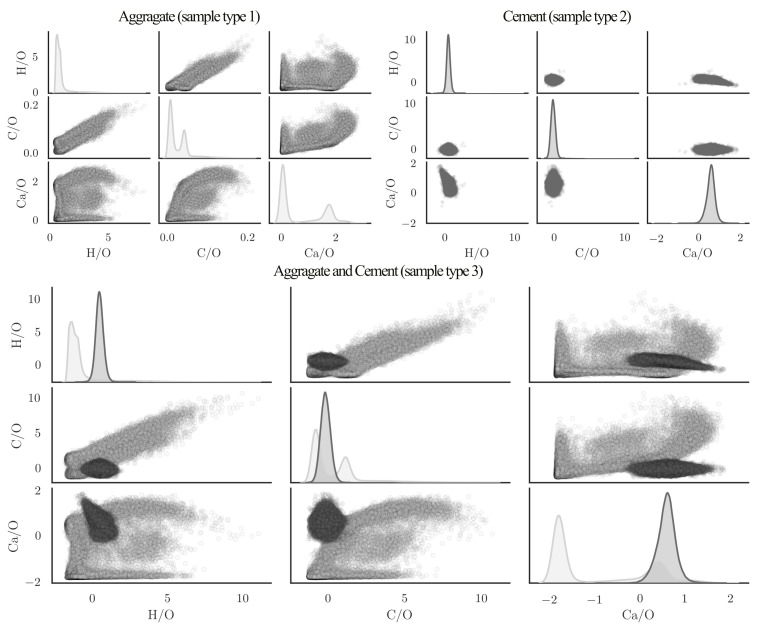
Pairwise plot of scaled virtual concrete in VIS-NIR range.

Figure 4 illustrates different elements in pairwise plots in which the diagonal plots are representing the distribution of data with kernel density estimation (KDE) function. The elements of the hydrated cement are homogeneously distributed and as a result the derived element ratio appear concentrated within the given plots. On the contrary, aggregates have a wide distribution of elements and their content and therefore the evaluated element ratios scatter in different degrees (see Figure 4).

Since cement consists essentially of burnt stone, such as limestone, clay, and dolomite, aggregates and cement show a large overlap in the element distributions. The comparison of density distributions derived from the measurement results show this. A comparison of these functions in relation to cement and aggregate illustrates the difficulties of differentiation. It is easy to discern between cement and aggregates in the areas, where the intensity of aggregates is lower or higher than minimum or maximum intensity of cement. The material allocation of the measuring points, where the intensity lies in this overlapping area, requires special attention in order to be able to determine whether the material measured is cement or aggregates. Due to overlapping of the common elements, it may not be sufficient to use a single distinguishing element and therefore several elements should be considered simultaneously. These findings are in good agreement with the literature study of element distribution in minerals discussed in the previous section.

### 3.2. Model Training and Verification

**Figure 5:**
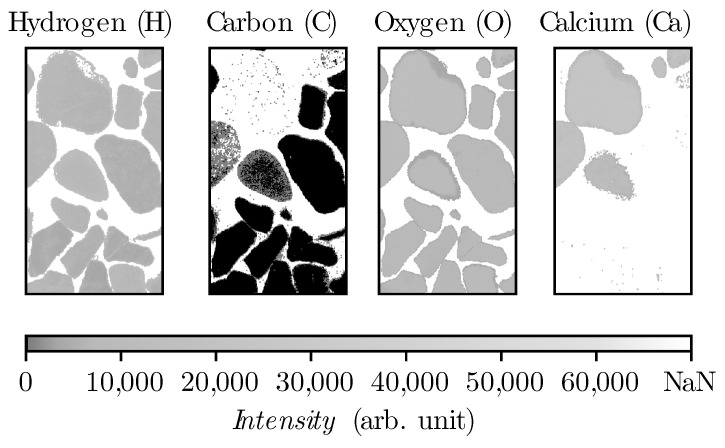
Gravel heatmap after epoxy removal from sample type (1) in spectral range 525–940 nm.

To train the algorithm a virtual concrete consisting of the two data-sets of sample type (1) and (2) have been measured and labeled. The first data set represents aggregates and the second one hydrated cement paste. From the sample type (1), the epoxy resin has been removed with thresholding the intensity for each element (H, C, O, Ca) as is shown in Figure 5.

**Figure 6:**
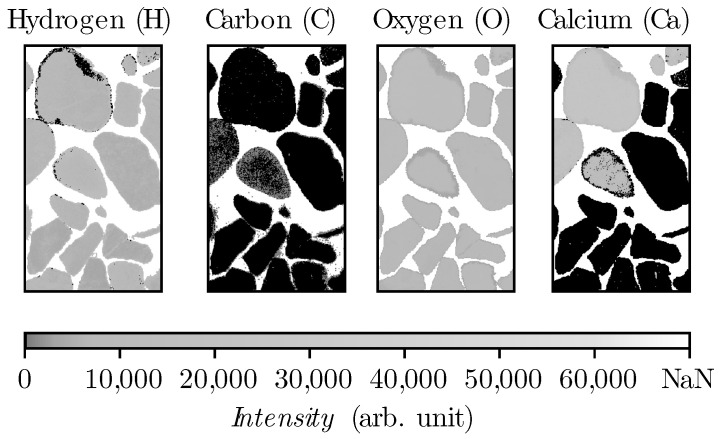
Masking oxygen heatmap regarding the empty points within the aggregates after epoxy removal from sample type (1) in spectral range 525–940 nm.

In order to avoid losing data points after data removal linked to epoxy, the empty points within the aggregates have been filled up with the value of zero, by masking the oxygen layer to them (see Figure 6). By this procedure, the aggregates without hydrogen, carbon or calcium will also be detected. To avoid biasing in training, these data-sets have been leveled equally to the size of 74,000 samples in each set by random selection. These two sets were merged and shuffled together.

In this study, the prepared data for learning is labeled as cement/aggregate and therefore, supervised learning with classification algorithms has been used. An advantage of supervised learning is the possibility of measuring the accuracy of the trained model with the test data set. The virtual concrete is not only used for training purpose but it has also been used for tracking the predictions and determine the accuracy of the model.

The virtual concrete has been divided randomly into two sets for training and for testing with the size of 118,400 and 29,600, respectively.

**Figure 7:**
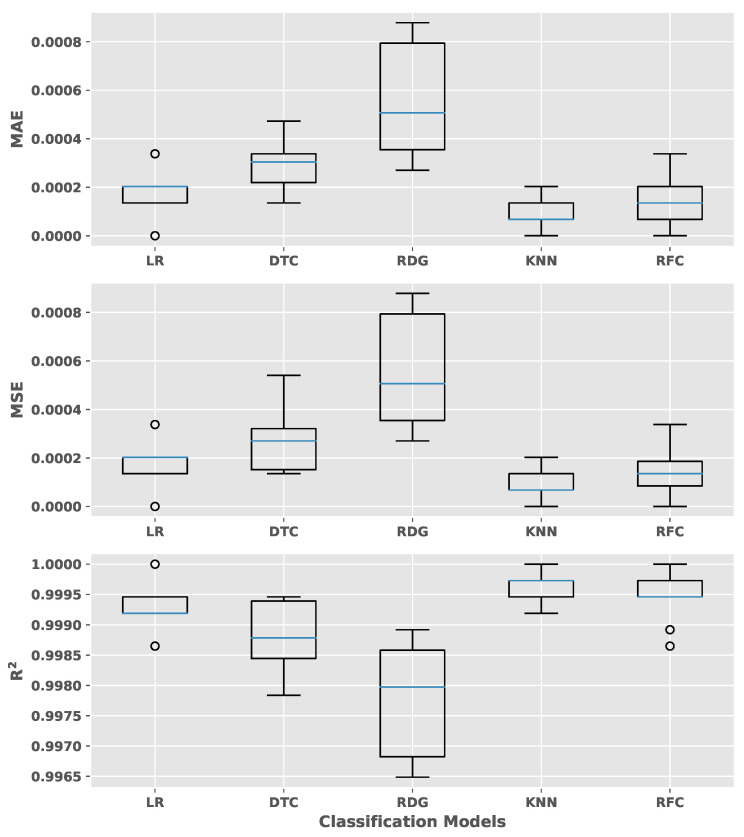
An algorithm comparison with cross-validation method with 10 folds. The mean absolute error (MAE), mean squared error (MSE), and the coefficient of determination ($R^{2}$) of different algorithms are shown.

A cross-validation for the evaluation the performance of the model has been performed and compared with different algorithms. The kNN algorithm showed an outstanding performance among Logistic Regression, Decision Tree, Ridge, and Random Forest. Mean absolute error (MAE), mean squared error (MSE), and the coefficient of determination ($R^{2}$) scoring metrics have been evaluated to check the effect of the outliers on performance (see Figure 7). The kNN algorithm has shown the highest value in all metrics compared to the other algorithms.

**Figure 8:**
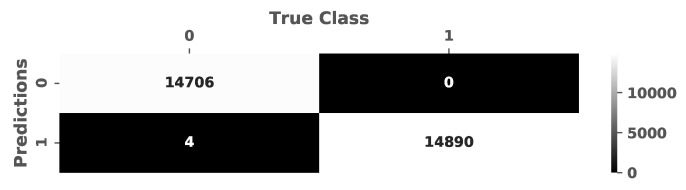
The confusion matrix of the test sets.

A testing of the trained model was performed with different test data sets from type 1 samples, consisting of aggregate and resin. The evaluation of these data showed a high accuracy with a recall value above 99% as the predicted and true class values are illustrated in the confusion matrix (see Figure 8).

**Figure 9:**
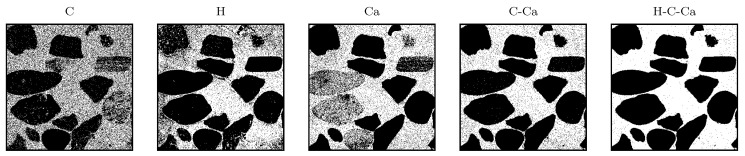
Training iterations to find the optimized setting for training the model and the quality of segregation in practice.

Since the developed model should be applicable for in situ concrete, it was very important to verify the predictions also for concrete. In a second step the model was verified with concrete samples (sample type (3)). The quality of the material differentiation was then checked visually, which was possible without much effort due to the grain size of the aggregate being between 8 and 16 mm. It is expected that the entire area between the aggregates will be detected as cement, as no sand was added to the concrete mixture. To train the model, different variations of elements have been tried in an iterative process until the segregation has been completed visually. Here, as before, it became apparent that no satisfactory result could be achieved by considering only the information from one element. Since in sample type (3), there were only aggregates size from 8 to 16 mm, it is expected that the algorithm shows a complete hollow space among aggregates. As Figure 9 shows five iterations with constant kNN parameters (k = 3) and modification in data frame, employing single elements such as carbon, calcium, or hydrogen, it is not possible to distinct completely the aggregates from cement paste. And there are always some remaining particles misclassified as aggregates in cement. This is very crucial to be sure that no cement has been misclassified, because normally, concrete also contains sand with a particle-size distribution between 4 and 0.063 mm and also fine particles smaller than 0.063 mm. According to [24], it is not possible to truly measure the particles smaller than the focal spot of the laser or partially ablated cement and aggregate, which in our case is 80 μm. However, it is important that no particle larger than the focal spot is misclassified. Since the *kNN* algorithm functions with distance metrics, usually based on euclidean geometry, the intensity of each element is a vector in this function. As it is illustrated in Figure 4, there are always measured points, where the intensity of aggregates are in between the range of minimum and maximum intensity of cement. By adding dimension to the vector with employing different elements, this minimizes the chance to have a point inside the multidimensional intensity matrix where cement and aggregate are close to each other.

This is illustrated in Figure 9, where the inclusion of several elements in the multidimensional vectors leads to a higher accuracy of the aggregate detection. Thus the quality of the aggregate exclusion could be improved significantly. Firstly, the model has been trained exclusively with carbon data. As it has found to be the element with the highest deviation in quantity over aggregates and cement. Furthermore, it is present in considerable quantities. It was expected that with hydrogen it would be possible to distinguish between aggregate and cement. The reason is that no hydrogen atom has been found in the minerals that form the aggregates and hydrogen is chemically bound only in hydrated cement. The developed model shows with involving element combination an improvement of the quality in distinction. This effect cannot only be observed statistically but also visually. The complete model in this study includes the combination of all calcium, carbon, hydrogen, and oxygen elements.

### 3.3. In Situ Sample

**Figure 10:**
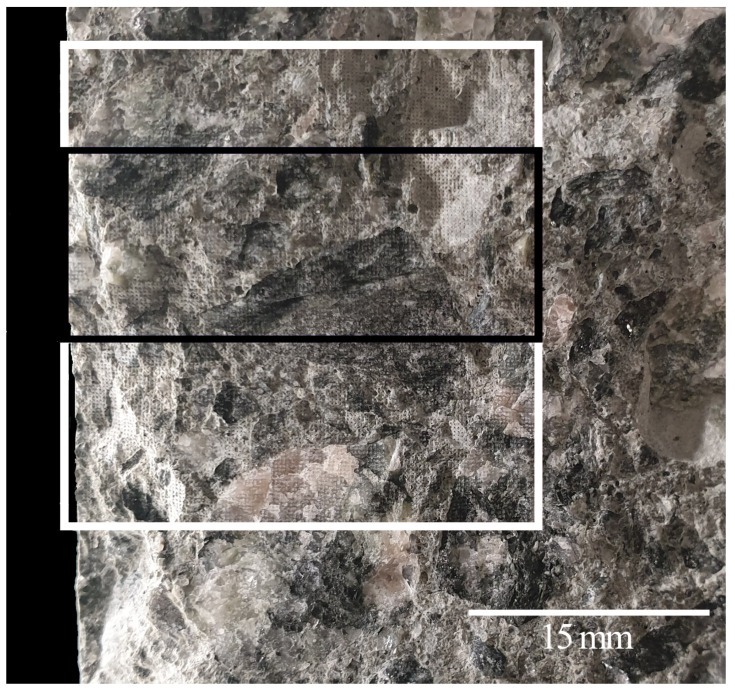
A specimen from an existing structure exposed to chloride, side of impact for the undamaged surface from the left-hand side and in addition through the depth of the concrete cover. The white rectangular shows the area that scanned by LIBS. The black rectangle marks the area for the determination of the chloride profile.

In Figure 10 the measuring surface of the drilled concrete core is shown. To quantify the chloride content within the hardened cement paste of that sample, it is required that the surface gets scanned with the LIBS method and the chlorine emission line is evaluated.

**Figure 11:**
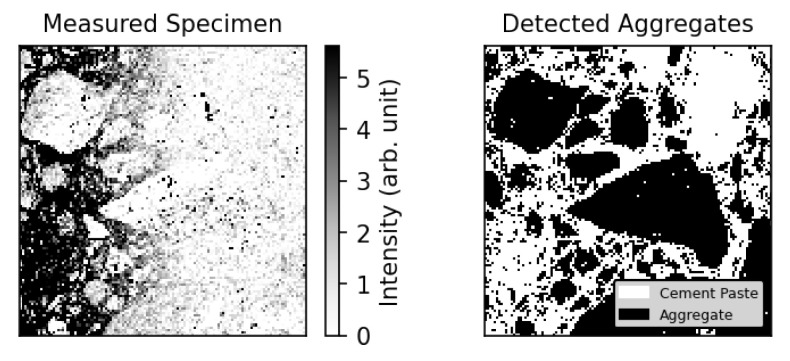
(**Left**) Heatmap from the chlorine channel of the scanned area (see Figure 10). (**Right**) Detected aggregates with Aggex.

In the determined chlorine heatmap of the specimen (see Figure 11 Left), there is a saturated dark area at the surface (high concentration), which fades away with ongoing depths. This dark area represents the chloride content. To evaluate the chloride content in cement paste, Aggex ran over the LIBS data set of the specimen to detect the aggregates and map it to the chlorine channel (black areas). Although the training data had a different origin, Aggex identified the aggregates of this sample satisfactorily.

## 4. Discussion

The application of the developed algorithm is demonstrated on a concrete core drilled from an existing reinforced concrete structure. Before applying this method, the trained aggregate extractor (Aggex) model was serialized and deployed in the software. This gives an unprecedented advantage over clustering algorithms such as k-mean as studied by [16]. To distinguish aggregates, no further processing on newly measured specimen is required. Unsupervised evaluation does not rely on external information, and therefore, a new process on each measured specimen is required which is time consuming and the misclassifications are not countable.

**Figure 12:**
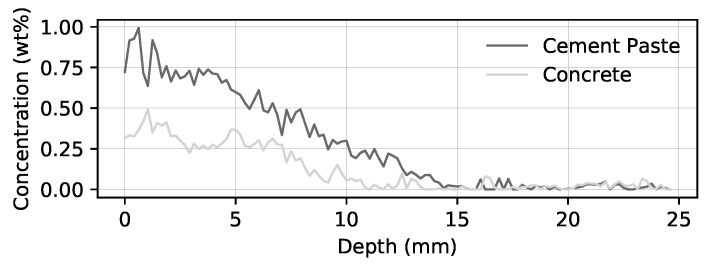
Comparison of depth dependent chloride content (chloride profile) of an in situ specimen with and without consideration of aggregates.

With the outlined approach the mean chloride content within a certain depth can be calculated in wt.-% based on the cement enabling the output of so-called chloride profiles (see Figure 12). This profile is generated by averaging all measurement spots of a specific depth of the specimen. The width of the area of interest for the calculation of the profile could be adjusted dynamically. So susceptible areas can be investigated in post-processing. That is an advantage compared to the conventional methods. LIBS makes it possible to dynamically observe the element content at different positions, widths, and depths of the specimen. With this data it is possible to determine more reliable chloride profiles for further evaluations.

One can see that the curve becomes smoother with gravel exclusion and that there is a shift to higher concentrations. With this data, more reliable information linked to the prediction of service life can be achieved compared to conventional methods. This enables more optimized maintenance and repair actions over service life as well as improving the sustainable use of structures. Furthermore, by also providing very localized information, a better understanding of chloride distribution in cracks can be achieved. This is important for future investigations as the cracks directly exposed to chlorides are very severe, causing extensive repair costs. As an example, the decks of parking garage should be mentioned.

## 5. Conclusions

Concrete is a heterogeneous material that is broadly used in construction. Chlorides from deicing salt or seawater may heavily influence the durability of reinforced concrete structures as it may cause corrosion and subsequently affect the service life. As relevant limit values linked to harmful substances such as chloride are specified in relation to the cement mass uncertainties may occur due to the lack of knowledge of the concrete compositions within the sample examined. By using the laser-induced breakdown spectroscopy technique element distributions of the scanned surface can be given. The acquired data from this technique needs further processing to quantify relevant element contents in materials that are examined. Furthermore, the data can be used for segregating the aggregates from the cement. A machine learning algorithm with LIBS data has been accomplished for this purpose. The model designed in this paper is based on a kNN algorithm with an accuracy close to 100%. To train the aggregate extractor four elements have been selected for stabilizing the accuracy, which are calcium, carbon, hydrogen, and oxygen. Oxygen has been used for internal normalization and the other elements mentioned for automated distinction. Cement and aggregate components can be very close to each other within its element distribution, which leads to huge overlapping in element intensities measured with LIBS. Despite high overlap in some elements such as calcium in cement and aggregate, with multivariate analysis it is possible to differentiate between aggregate and cement. As LIBS also provides a dynamic continuous observation over the scanned area of the specimen this enables achieving detailed information within a fast measuring process. The possibility to obtain locally very detailed information as well as rather global information (e.g. mean penetration depth) is important to design suitable maintenance and repair strategies for reinforced concrete structures. This enables more optimized actions linked to maintenance and repair over service life and therewith improving the sustainable use of structures.

## Figures and Tables

**Table 1:** Chemical oxides in aggregates and cement type I composition [12,13,14,15].

Chemicals	Basalt	Limestone	Sandstones	Granite	CEM I
SiO_2_	✓	✓	✓	✓	✓
Al_2_O_3_	✓	✓	✓	✓	✓
Fe_2_O_3_	✓	✓	✓	✓	✓
FeO	✓		✓	✓	
MgO	✓		✓	✓	✓
MgCO_3_		✓			
CaO	✓		✓	✓	✓
Na_2_O	✓	✓	✓	✓	✓
K_2_O	✓	✓	✓	✓	✓
CaCO_3_		✓			
TiO_3_			✓	✓	
P_2_O_5_	✓	✓	✓	✓	
MnO	✓		✓	✓	

**Table 2:** Element distribution weight percentage (wt.%) of major minerals that compose aggregates and cement type I (empty spaces mean no or a negligible content).

Element	Plagioclase		Quartz		Hematite		Magnetite		Amphibole		Pyroxene		Olivine		Mica		Calcit	CEM I	CEM I
	Min	Max	Min	Max	Min	Max	Min	Max	Min	Max	Min	Max	Min	Max	Min	Max		Clinker	Hydrated
Ca		14	11		35				10	40	47	34							
O	46	49		53		70	28	28	37	58	36	48	31	45	28	50	48	35	50
Si	20	32		47					22	34	21	28	14	20	7	29		9	7
Fe						30	72	72		39		42		55		41		5	3
Mg										22		24		35		18			
Al	10	19										13				28		4	3
K	14	14								6						10			
F																10			
Na		9								10		11				6			
H																1			3
Li										7						4			
Ti																7			
S																8			
C																	12		

**Table 3:** Selected emission lines.

Element	Wavelength (nm)
Hydrogen (H)	656.2
Carbon (C)	833.5
Oxygen (O)	844.6
Calcium (Ca)	849.8

**Table 4:** Standard deviation of selected elements in aggregates.

Element	C	H	Ca	O
Std	1.2	0.58	0.47	0.38

## Data Availability

To be released on github.
